# Long Term Outcomes of Anti-COVID-19 Vaccines in Patients with Systemic Lupus Erythematosus: A Multicentre Study

**DOI:** 10.3390/vaccines13070735

**Published:** 2025-07-08

**Authors:** Giovanni Benanti, Giuseppe A. Ramirez, Tommaso Schioppo, Lorenza Maria Argolini, Gabriella Moroni, Grazia Bonelli, Renato Alberto Sinico, Federico Alberici, Federica Mescia, Luca Moroni, Gabriele D. Gallina, Biancamaria Venerandi, Francesco Tamborini, Chiara Bellocchi, Lorenzo Beretta, Roberto Caporali, Enrica Bozzolo, Lorenzo Dagna, Maria Gerosa

**Affiliations:** 1 Unit of Immunology, Rheumatology, Allergy and Rare Diseases, IRCCS Ospedale San Raffaele, 20132 Milan, Italy; benanti.giovanni@hsr.it (G.B.); moroni.luca@hsr.it (L.M.); gallina.gabriele@hsr.it (G.D.G.); venerandi.biancamaria@hsr.it (B.V.); bozzolo.enrica@hsr.it (E.B.); dagna.lorenzo@hsr.it (L.D.); 2 Unit of General Medicine and Advanced Care, IRCCS Ospedale San Raffaele, 20132 Milan, Italy; 3 SMILE (Milan Lupus Cohort), via Olgettina 60, 20132 Milan, Italy; tommaso.schioppo@asst-santipaolocarlo.it (T.S.); lorenza.argolini@hotmail.it (L.M.A.); chiara.bellocchi@unimi.it (C.B.); lorberimm@hotmail.com (L.B.); roberto.caporali@unimi.it (R.C.); maria.gerosa@unimi.it (M.G.); 4 Faculty of Medicine, Università Vita-Salute San Raffaele, 20132 Milan, Italy; 5 Unit of Rheumatology, ASST Santi Paolo e Carlo, 20132 Milan, Italy; 6 ASST Pini CTO, Lupus Clinic, Division of Clinical Rheumatology, 20122 Milan, Italy; 7 Department of Biomedical Sciences, Humanitas University, IRCCS Humanitas Research Hospital, 20072 Milan, Italy; gabriella.moroni@hunimed.eu; 8 Renal Unit, Department of Medicine and Surgery, Università degli Studi di Milano Bicocca and ASST-Monza, 20900 Monza, Italy; g.bonelli2@campus.unimib.it (G.B.); renato.sinico@unimib.it (R.A.S.); 9 Department of Medical and Surgical Specialties, Radiological Sciences and Public Health, University of Brescia, 25121 Brescia, Italy; federico.alberici@gmail.com (F.A.); federica.mescia@gmail.com (F.M.); 10 Fondazione Ca’ Granda IRCCS Ospedale Maggiore Policlinico Milano, Divisione di Nefrologia e Dialisi, 20122 Milan, Italy; francesco_tamborini@asst-pavia.it; 11 Fondazione IRCCS Ca’ Granda Ospedale Maggiore Policlinico di Milano, Referral Centre for Systemic Autoimmune Diseases, 20122 Milan, Italy; 12 Department of Clinical Science of Community Health, Section of Internal Medicine, Università degli Studi di Milano, 20122 Milano, Italy; 13 Department of Clinical Science of Community Health and Research Center for Adult and Pediatric Rheumatic Diseases, Università degli Studi di Milano, 20122 Milano, Italy

**Keywords:** systemic lupus erythematosus, COVID-19, vaccines, long-term, adverse events

## Abstract

**Introduction**: Systemic lupus erythematosus (SLE) is associated with infection-related morbidity. The risk of adverse outcomes secondary to infections was exacerbated during the recent COVID-19 pandemic, prompting mass vaccination with the novel mRNA-based and viral-vectored vaccines. Limited data exist on the long-term impact of vaccination in patients with SLE. **Methods**: A post-vaccine group (PVG, *n* = 284) from a multicentric cohort of vaccinated patients with SLE from six tertiary referral centres in Northen Italy was compared with a control group (CG, *n* = 223) of similar demographics observed in the 2015–2019 period to investigate survival, hospitalisation, pregnancy, disease flare, disease progression, infection, and chronic complication accrual rates. **Results**: We did not observe excess SLE flares, SLE progression, hospitalisation, or pregnancy complications in the PVG. Cardiovascular complications due to comorbidities or to SLE were lower in the PVG than in the CG. Infections were more frequent in the PVG, related to COVID-19 in half of the cases, and were associated with SLE flares. **Conclusions**: Taken together, these data indicate that anti-COVID-19 vaccines are safe in the long-term when administered to patients with SLE. Stable, non-null rates of chronic comorbidity accrual and hospitalisation point out the existence of persistently unmet needs in patients with SLE.

## 1. Introduction

The recent pandemic of severe acute respiratory syndrome coronavirus 2 (SARS-CoV-2)-related disease (COVID-19) posed unprecedented challenges to our society, fuelling ongoing dynamics in communication, politics, and economics, highlighting existing flaws in welfare systems, and synergising with other global crises to affect individual prospects for the future, in addition to physical morbidity and mortality [1,2,3]. Patients with chronic disorders, including systemic autoimmune diseases, proved particularly vulnerable to the impact of the pandemic due to enhanced susceptibility to infections and their complications. Disruption of healthcare systems and accumulation of delays in waiting lists for first or control clinical assessments further exacerbated the pandemic burden for complex patients with one or more causes of morbidity [4,5,6].

Patients with systemic lupus erythematosus (SLE) were found at higher risk of COVID-19-related complications, especially in cases of higher-intensity immunosuppression with glucocorticoids [7,8,9]. Later studies revealed that at least part of this risk might be explained by the disproportionally high frequence of natural anti-type I interferon autoantibodies [10], a known risk factor for severe COVID-19 [11]. Indeed, infections, including COVID-19, are a major cause of morbidity and mortality for patients with SLE and constitute a frequent trigger for disease onset or exacerbation [12,13]. Accordingly, active immunisation through vaccines is highly recommended by national and international guidelines for SLE and other connective tissues diseases [14].

Vaccination played a crucial role in the resolution of the COVID-19 crisis. In fact, the rapid development of highly effective novel vaccination technologies such as mRNA vaccines and viral-vectored vaccines enabled active and widespread immunisation of the population, minimising the risk of unfavourable outcomes for vulnerable subjects and reducing the contagion chain [15,16,17]. Acute and chronic morbidity is significantly more frequent with COVID-19 than with anti-COVID-19 vaccines [18,19,20,21]. Nonetheless, the unprecedented, almost synchronic administration of these new drugs in the whole human population revealed the existence of a panel of potential vaccine-associated adverse events [19,22,23]. While the size of the vaccinated population was sufficiently high to justify the coincidental occurrence of the vast majority of these events as just the expression of basal risk rates [24], misinformation and the viral spread of unverified data synergised with existing trends of mistrust in public and/or academic institutions to foster vaccine hesitancy or fear in the general population [25,26,27,28].

This issue was particularly relevant in selected groups, such as those of patients with immune-mediated disorders. While being underrepresented in, or excluded from, vaccine registration trials, these patients were also potentially more prone to the development of adverse events potentially linked to dysfunctional immune response [29,30]. Observational studies from global registries or homogeneous cohorts have progressively filled this gap, providing initial evidence of short-term safety for anti-COVID-19 vaccination in multiple disease settings, including allergic and autoimmune disorders [31,32]. Limited data exist regarding potential adverse events occurring in the long-term in patients with rheumatic musculoskeletal diseases. To address this issue, we elected SLE as a paradigm of systemic autoimmune disorders and performed a long-term follow-up study in a cohort of vaccinated patients with SLE who were previously analysed in close temporal relation to the receipt of their first doses of anti-COVID-19 vaccines [33].

## 2. Materials and Methods

We leveraged on a well-characterised multicentre cohort of patients classified with SLE according to the EULAR/ACR 2019 criteria [34] and undergoing anti-COVID-19 vaccination between December 2020 and October 2021 [33]. This cohort comprises six tertiary referral centres for the treatment of systemic immune-mediated diseases, namely Lupus Clinic at Unit of Immunology, Rheumatology, Allergy and Rare Diseases, IRCCS Ospedale San Raffaele, Milan; Lupus Clinic, Clinical Rheumatology Division of ASST Pini-CTO, Milan; Renal and Rheumatology Units, San Gerardo Hospital, Monza; IRCCS Humanitas Research Hospital; ASST Spedali Civili, Brescia, Italy; and Referral Center for Systemic Autoimmune Diseases, Fondazione IRCCS Ca’ Granda Ospedale Maggiore Policlinico, Milan. Retrospective follow-up data were collected until May 2024 upon written informed consent under the following protocols: “MLC protocol” (IRCCS Ospedale San Raffaele, ASST Pini CTO, Fondazione IRCCS Ca’ Granda Ospedale Maggiore Policlinico, Ethics Committee Comitato Etico Milano Area 2; approval number 0002450/2020 and San Raffaele Hospital Ethics Committee, approval number 84/INT/2019), protocol NEF0032023 (IRCCS Humanitas, approved by the Ethics Committee of the same institution), protocol “Coorte MaRe” approved by the ethics Committee of ASST Spedali Civili di Brescia *n* 4945 (ASST Spedali Civili), and protocol 1093/2021 (San Gerardo Hospital, Monza). Data sharing among centres occurred conforming to the European Union prescriptions for data protection [35]. Patients belonging to the original cohort were classified as members of a post-vaccination group (PVG). In parallel, we retrieved data from a control group (CG) consisting of a cohort of patients with SLE fitting the same criteria and observed in the five years preceding COVID-19, as part of a larger observational protocol (“Pan-immuno”, approved by the IRCCS Ospedale San Raffaele Institutional Review Board with reference number 22/INT/2018), conforming to the Declaration of Helsinki.

### 2.1. Timeframes of Observation

Observations in the PVG started from the date of the first anti-COVID-19 to the last available follow-up visit in May 2024. In the CG, the observation timeframe started with the first visit performed in 2015 and ended with the last available visit up to December 2019. We also calculated the timing from each patient’s baseline observation to the development of complications of special interest, as defined below.

### 2.2. Short-Term Adverse Events

Data on short-term adverse events related to the first and second vaccine injections were retrieved from the original dataset [33] and were defined as any adverse event occurring up to one month after each injection.

### 2.3. Long-Term Complications of Special Interest

We assessed seven types of long-term complications of special interest: (1) death; (2) hospitalisation for any cause; (3) disease flares; (4) new SLE-related manifestations; (5) infections; (6) accrual of new chronic comorbidities; (7) pregnancy morbidity. Disease flares were defined as new-onset or relapsing SLE manifestations requiring medium/long-term treatment upgrade and/or causing hospitalisation or death. Temporary, self-resolving manifestations, even if potentially attributable to SLE, were recorded among the short-term adverse events (see above). We recorded whether, during the observation timeframe, patients developed SLE-related manifestations that were not part of the previous clinical history (e.g., new-onset lupus nephritis in a patient with no previous renal involvement). These manifestations were classified according to the nine domains of the 2004 British Isles Lupus Assessment Group (BILAG) index [36]. We recorded all infections causing loss of work/schooldays and/or requiring treatment with antimicrobial treatments and/or causing hospitalisations [13]. We defined four sets of chronic comorbidities of special interest, which were recorded if (a) their diagnosis had been formulated within and (b) the diagnostic work-up or the onset of potential symptoms had not started before the timeframe of observation. A first group of comorbidities consisted of common causes of morbidity and mortality in the general population and encompassed cardiovascular disorders other than hypertension, hypertension, cancer, neurological disorders, and pulmonary disorders. A second set included morbidity with potentially higher social and psychological implications: non-SLE cutaneous disorders, psychiatric disorders, and gonadal failure. A third group of comorbidities encompassed secondary autoimmune diseases such as Sjoegren’s syndrome, rheumatoid arthritis, systemic sclerosis, inflammatory myopathies, anti-synthetase syndrome, Hashimoto’s thyroiditis/Graves’ disease, and coeliac disease. The fourth disease subgroup included comorbidities that were more frequently associated with post-COVID-19 or long-COVID-19 or for which a potential association with anti-COVID-19 vaccines had been hypothesised [18,19,37,38,39,40]: arrhythmia, myocarditis or pericarditis, chronic urticaria/itching, and fibromyalgia. We recorded the number of pregnancies and of pregnancy complications occurring during the observation timeframe.

### 2.4. General Features

In addition to the follow-up variables of special interest, data were recorded on patients’ demographics, disease onset and general disease characteristics. The BILAG-2004 index [36] was used to estimate SLE extent in patients’ history and SLE activity at the start of the observation timeframe. The Definition Of Remission in SLE (DORIS) criteria were applied to define disease quiescence [41]. Finally, we collected data on immunomodulant and immunosuppressive treatments (including prednisone daily dose) at baseline and at time of the last visit within the observation timeframe.

### 2.5. Statistical Analysis

Data were analysed using Statacorp STATA^®^ v18.0 and the online OpenEpi tool (www.openepi.com) v3.01. Continuous variables are expressed as median and interquartile range, while categorical variables are expressed as number and percentage on the total population. Comparisons of continuous variables between groups were performed using the Mann–Whitney U-test. Incidence rates and categorical variables were compared by groups through the use of the chi-square tests with Fisher’s exact correction as appropriate. Cox regression was used to determine the potential impact of distinct variables on time-dependent outcomes.

## 3. Results

### 3.1. General Features

Follow up data were available from 284 of 452 PVG patients with SLE in the original cohort [33]. Most patients were women (89%) in their sixth decade (30%) and had a median disease duration of 18 (11–27) years at time of vaccination. The median observation timeframe (post-vaccine follow up) was 33 (31–35) months. Musculoskeletal and mucocutaneous domains were the most frequently represented (Supplemental Table S1). A total of 142 (50%) patients were taking immunosuppressants, 57 (20%) biotechnological immunomodulatory drugs (belimumab), and 164 (58%) were taking corticosteroids at time of vaccination. Among patients taking immunosuppressants, only six had biotechnological immunosuppressants; this subgroup was not further analysed due to its excessively small sample size. The majority of patients had been vaccinated with the Pfizer anti-COVID-19 vaccine (*n* = 243; 86%), while Moderna (*n* = 34; 12%), Astrazeneca (*n* = 6; 2%), and Johnson & Johnson (*n* = 1, <1%) vaccines were less represented. No difference in any of the study outcomes was observed when patients were stratified by specific vaccine. The percentage of patients receiving immunosuppressants and corticosteroids did not change significantly between the start and the end of the observation timeframe. PVG patients were compared with a cohort of 223 CG patients, who were observed for a median time of 32 (18–44) months. Their main clinical features are reported in Supplemental Table S2.

### 3.2. Short-Term Adverse Events Following Vaccination

Seventy-nine PVG patients (28%) had one or more adverse reactions after the first (*n* = 45; 16%) and second (*n* = 62; 22%) vaccine injections. The most frequent adverse reactions were fever (44%), arthralgia (24%), fatigue (20%), local reactions at site of injection (18%), myalgia (14%), gastrointestinal symptoms (14%), and headache (13%; Supplemental Table S3). A history of constitutional symptoms (OR = 2.24, 95% confidence interval, CI = 1.31–3.83; *p* = 0.003), treatment with belimumab (OR = 1.87, 95%CI = 1.02–3.46; *p* = 0.044), or conventional synthetic immunosuppressants (OR 1.73, 95%CI = 1.02–2.94; *p* = 0.040) at time of vaccination constituted risk factors for short-term adverse reactions.

### 3.3. Long-Term Complications

#### 3.3.1. General Outcomes

**Figure 1:**
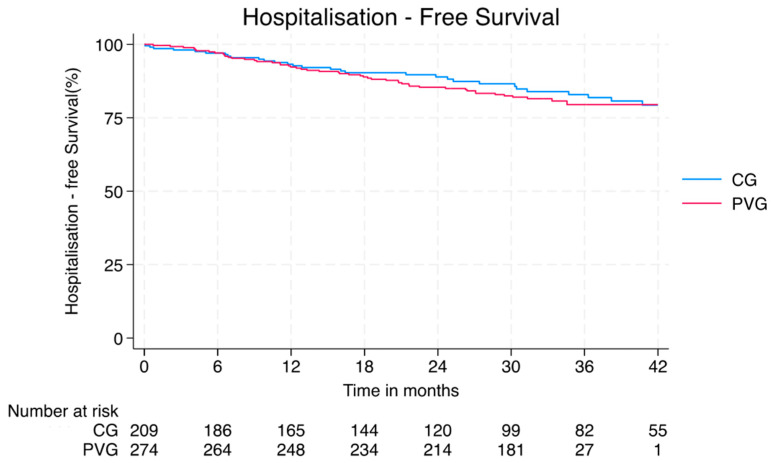
Kaplan–Meier curve and at-risk table for hospitalisation-free survival in the post-vaccine group (PVG; red) and the control group (CG; blue). No significant difference was observed between the two groups. CG–control group; PVG–post-vaccination group.

Three patients, two in the PVG and one in the CG, died during the observation timeframe. At the last visit, 211 (75%) patients in the PVG met DORIS remission criteria. There were 80 hospitalisations, affecting 61 patients in the PVG and 59 affecting 45 patients in the CG. Hospitalisation rates were similar in the PVG (1.59 events/100 person-months) and in the CG (1.16/100 person-months, *p* = 0.054; Figure 1). The most frequent cause of hospitalisation in the PVG was diagnostics [*n* = 18 (30%)] (mainly kidney biopsy)], followed by general surgery [*n* = 10 (16%)], orthopaedic surgery [*n* = 8 (13%)], and infections [*n* = 8 (13%)]. In the CG, the main causes of hospitalisation were infections [*n* = 11, (24%)], followed by cardiovascular events [*n* = 9 (20%)], general surgery [*n* = 7 (15%)], neurological events [*n* = 6 (13%)], and diagnostics [*n* = 5 (11%); Table 1].

In the PVG, similar frequencies of hospitalisations were observed among patients who experienced vs. who did not experience a short-term adverse event after any vaccination dose [16 (21%) vs. 45 (22%); *p* = 1.000]. Higher hospitalisation rates were observed among patients taking [34/112 (30%)] vs. not taking prednisone doses equal to or higher than 5 mg/day [27/168 (16%); *p* = 0.005]. No differences were found after stratification either by immunosuppressants, belimumab, BILAG domain involvement, or active BILAG domains at the time of vaccination.

#### 3.3.2. Infections

A total of 138 (49%) PVG patients reported at least one infectious episode, and 123 (43%) patients reported at least one COVID-19 infection in the follow-up period. Infection prevalence was lower in the CG (35%). Accordingly, infection incidence rate was greater in the PVG than in the CG (2.89 vs. 1.65/100 person-months; *p* < 0.001). Short-term adverse reactions to vaccines and disease activity at time of vaccination did not predict the eventual occurrence of infection in the PVG. Compared to patients not receiving immunosuppressants, patients with conventional synthetic immunosuppressants had a higher prevalence of infections [80/141 (57%) vs. 58/143 (41%); *p* = 0.009]. Patients taking belimumab had no significant difference on infection prevalence. Higher infection rates were also observed in patients with a history of renal involvement [100/184 (54%) vs. 38/100 (38%) in patients with no history of nephritis; *p* = 0.009].

#### 3.3.3. SLE Flares and Progression

**Figure 2:**
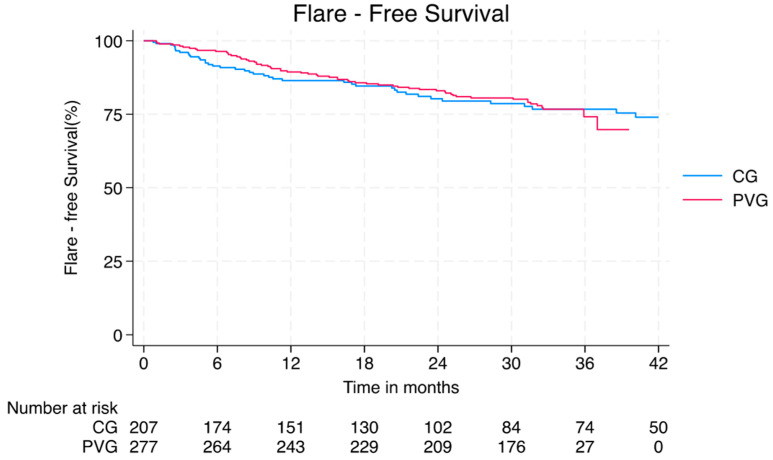
Kaplan–Meier curve for Flare-Free Survival in the PVG (red) and CG (blue). No significant difference was observed between the two groups. CG–control group; PVG post-vaccination group.

Post-vaccination flares occurred in 68/284 (24%) PVG patients, yielding a flare rate of 0.76/100 person-months. This was comparable to flares observed in the CG (0.90/100 person-months, *p* = 0.382; Figure 2). Compared to quiescent disease, higher flare rates were observed in patients with active constitutional [5/7 (71%) vs. 23/276 (63%); *p* = 0.010], musculoskeletal [13/32 (41%) vs. 55/251 (22%); *p* = 0.027], or mucocutaneous [ 8/17 (47%) vs. 60/266 (23%); *p* = 0.036] domain at time of vaccination. No differences were observed in terms of SLE flare rates when stratifying by corticosteroid, belimumab, or conventional immune suppressant treatment or by occurrence of short-term adverse reactions. Patients in the PVG who experienced at least one infection had an increased proportion of disease flares compared to patients with no infections [25/146 (17%) vs. 43/138 (31%); *p* = 0.008].

**Figure 3:**
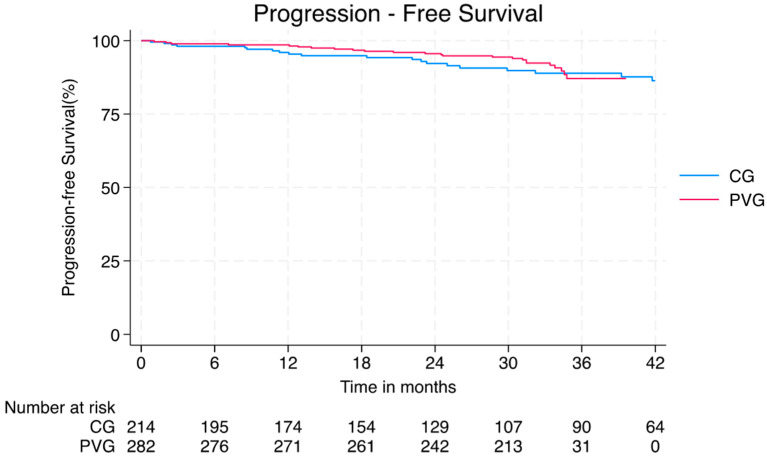
Kaplan–Meier Curve for Progression-Free Survival in the PVG (red) and CG (blue). No significant difference was observed between the two groups. CG–control group; PVG–post-vaccination group.

Disease progression with the development of active disease in unprecedented BILAG domains was observed in *n* = 26 (9%) of subjects in the PVG and *n* = 34 (15%) in the CG, yielding comparable incidence rates (0.30 vs. 0.50/100 person-months; *p* = 0.063; Figure 3). The most frequent domains involved in the progression were musculoskeletal and mucocutaneous for the PVG and cardiopulmonary and haematologic for the CG (Table 2). Accordingly, PVG patients had more new musculoskeletal manifestations [*n* = 17 (6%) vs. *n* = 3 (1%); *p* = 0.010] but significantly less haematological manifestations [*n* = 0 (0%) vs. *n* = 12 (6%); *p* < 0.001) and cardiopulmonary manifestation [*n* = 3 (1%) vs. *n* = 11 (5%); *p* = 0.012] than patients in the CG. No differences among PVG patients were observed based on therapy, short-term adverse events, infections history, disease history, or active disease domain at the time of vaccination.

#### 3.3.4. Chronic Comorbidities

A total of 34 PVG patients accrued one or more new chronic comorbidities during the observation timeframe. The most frequent cause of morbidity was cancer (*n* = 10 patients, 4%), followed by cardiovascular disorders (*n* = 6 patients, 2%), end-stage kidney disease (*n* = 5 patients, 2%), other autoimmune-diseases (*n* = 5 patients, 2%), and pulmonary disorders (*n* = 4 patients, 1%). Most new cancer diagnoses (7/10) were performed in the 2021–2022 period. No difference in new chronic comorbidities prevalence was observed based upon the use of immunosuppressants, belimumab, or corticosteroids or based on any short-term adverse reactions. A higher frequency of new chronic comorbidities was observed in patients without a history of cardiopulmonary manifestations [27/216 (13%) vs. 2/68 (3%); *p* = 0.021] or patients with active haematological manifestations [6/25 (24%) vs. 23/259 (9%); *p* = 0.030].

No excess rates of new chronic comorbidity accrual were observed in the PVG compared to the CG. The CG had an increased rate of new cardiovascular diseases (0.35 vs. 0.07 per 100-person month; *p* < 0.001), neurological disorders (0.09 vs. 0/100 person-month; *p* = 0.007), psychiatric disorders (0.08 vs. 0/100 person-months; *p* = 0.016), and fibromyalgia (0.05 vs. 0/100 person-months; *p* = 0.082; Table 3).

#### 3.3.5. Pregnancies

During the timeframe of observation, we recorded 11 pregnancies in 9 subjects in the PVG and 16 in 15 subjects in the CG (Table 4). Among the PVG, one patient developed pre-eclampsia, one patient developed a chorioamnionitis, and one developed lupus nephritis. Except for one spontaneous abortion in the first trimester and one infant diagnosed with 18th chromosome trisomy, all other pregnancies had favourable foetal outcomes. In the CG, pregnancy complications were recorded in six cases, with three spontaneous abortions, one pre-term delivery following CMV infection, one case of lupus nephritis, and one case of non-infectious endocarditis. No significant differences were observed. No difference in pregnancy number and pregnancy-related adverse event was observed based on therapy, short-term adverse events, or disease history at vaccination.

## 4. Discussion

In this multicentre study focusing on the long-term health status of patients with SLE having received anti-COVID-19 vaccines, we observed relatively low rates of complications following the 2020–2021 campaign where the novel mRNA and viral-vectored vaccine platforms were introduced. Furthermore, we did not detect significant changes in survival, hospitalisation rates, disease stability, accrual of new chronic causes of morbidity, and pregnancy outcomes in comparison to the pre-COVID-19/pre-vaccine era. Patients experiencing short-term adverse events to anti-COVID-19 vaccination had no higher risk of long-term morbidity or adverse outcomes. Higher baseline activity and/or receiving deeper immune suppression conferred a higher risk of flares and hospitalisation. Higher infection rates were calculated in the cohort observed during the COVID-19 pandemic compared to the CG, especially in subjects taking immunosuppressants or with a history of lupus nephritis. Indeed, most PVG patients reporting one or more infections had COVID-19.

A crucial principle in vaccinology is the ability of vaccines to uncouple antimicrobial protection through immunisation from infection morbidity. Aberrant deployment of the immune system in SLE and other immune-mediated disorders has historically prompted conjectures about potential risks of unsolicited vaccine-induced inflammation in patients with these disorders. In line with consolidated evidence with other vaccine platforms [42,43,44], our data show that anti-COVID-19 vaccines do not associate with excess SLE flare rates in the long-term [45], nor with disease progression and involvement of unprecedented BILAG domains. These data suggest that anti-COVID-19 vaccines do not induce persistent perturbations of the immune response in patients with SLE. In parallel, we observed that patients in the PVG did not experience higher rates of accumulation of new chronic comorbidities compared to the CG. New cancers were the most frequent comorbidities, and, while the overall incidence was comparable between the two groups, they were mainly diagnosed (7/10) in 2021–2022. This is consistent with an observed delay in cancer screening and diagnosis at the 2020 peak of the pandemic, due to disruptions of healthcare services [46,47]. Interestingly, we observed lower rates of new cardiovascular comorbidities and/or SLE-related manifestations in the PVG compared to the CG. Furthermore, patients with established cardiopulmonary morbidity had lower rates of further morbidity accrual. These data might subtend improved control and prevention of cardiac and pulmonary conditions in patients with more recent follow up, possibly also due to changes in practice and scientific knowledge secondary to the COVID-19 pandemic [48,49].

Despite the established role of infectious agents (especially of viral origin) in triggering SLE flares [12,13,50], vaccination rates in patients with SLE remain suboptimal [9,25,26,27,51,52]. While part of this phenomenon might be attributed to insufficient knowledge sharing among tertiary and primary settings of care for patients with SLE [50], significant barriers are also constituted by the spread of misinformation through social media platforms and mass-media, along with widespread loss of trust in academic/public institutions [53]. Lack of long-term safety data about the new mRNA-based or viral-vectored vaccines due to their emergency approval at the peak of the COVID-19 pandemic has further fuelled vaccine hesitancy. Our data suggest that, in line with consolidated knowledge with other vaccines [54], anti-COVID-19 immunisation does not associate with safety signals of concern in the short- [55,56,57,58,59,60,61] and long-term in vulnerable subjects, such as patients with SLE. Conversely, infections remain a major cause of morbidity in patients with SLE and other rheumatic musculoskeletal diseases, especially in case of deep immunosuppression [16]. In line with previous reports [62,63], we indeed observed that immunosuppression was a major risk factor for vaccine failure and development of clinically overt COVID-19. COVID-19 contributed to the relatively higher rate of infections observed in the PVG compared to the CG. Infections, in turn, were associated with higher rates of SLE flares. While supporting a model of SLE as a disease with generalised dysfunction of the immune response [13,64], this evidence confirms that vaccination might possibly prove as crucial as immunosuppression in preventing disease-related complications, being them either autoimmune phenomena or simply infections.

Patients in the PVG had similar hospitalisation rates to patients observed in the pre-COVID-19 era; in line with previous reports, about 10% of patients required hospitalisation at least once over the course of one year [65]. While this finding is further reassuring in terms of the potential impact of anti-COVID-19 vaccination in patients with SLE, it might also suggest that improvements are still warranted for an optimal management of patients’ needs. Indeed, worsening trends in the socioeconomical stability of healthcare systems, with potential overload of hospital-based care due to insufficient development of local facilities [66,67,68], might exacerbate existing limitations to the early detection and tackling of clinical destabilisation in frail patients, including patients with SLE [69].

Globally, our results indicate that anti-COVID-19 vaccines do not add to the baseline risk of morbidity of patients with SLE. However, multiple limitations should be considered for an appropriate interpretation of these data. First, this study focused on a distinct, though paradigmatic, autoimmune disease, restricting the potential generalisability of our findings to other immune-mediated diseases. Second, data collection was retrospective, which introduced a potential risk of underreporting for minor manifestations. This might have been further exacerbated by heterogeneity between the PVG and the CG, with the latter being observed in a more remote timeframe. Third, we did not collect data about the number of booster doses effectively received by the patients after the first two injections. Similarly, we had no information about vaccine coverage for non-SARS-CoV-2 pathogens, preventing a comprehensive analysis of factors conferring susceptibility to infections in our study cohorts. Moreover, while no difference was observed among specific vaccine platforms, the small sample size of non-mRNA-based vaccines prevents accurate conclusions about their specific long-term safety profile. Fourth, in the absence of experimental data about antibody and/or T-cell responses after vaccination, we were unable to correlate post-vaccine COVID-19 infection rates with quantitative measures of effective immunisation. Similarly, since no precise data on the timing and type of infections were available, we could not establish whether COVID-19 carries a different risk profile for inducing disease flares compared to other infections. Therefore, this long-term analysis is limited to safety assessment. Last, although we did not observe significant changes in pregnancy rates and in the frequency of pregnancy morbidity, we did not have complete information on pregnancy intention among PVG and CG patients in their fertile age, preventing the acquisition of definite data about fertility.

## 5. Conclusions

Our data suggest that anti-COVID-19 vaccines are safe in patients with SLE and do not adversely impact the disease course, pregnancy, or the overall health status. Since infections are known to precipitate disease flares, our data add to the evidence that vaccinations have a beneficial impact on SLE patients and support practices aiming at mitigating vaccination hesitancy. Further studies in other cohorts are warranted to confirm our results and/or extend their applicability to other chronic diseases.

## Figures and Tables

**Table 1:** Specific causes of hospitalisation. Percentages of each specific cause are referred to the total number of hospitalised patients. Some patients had multiple hospitalisations. CG–control group; PVG–post-vaccination group.

Hospitalised Patients by Cause of Hospitalisation: *N* (%)	CG (*n* = 45/223)	PVG (*n* = 61/284)	*p*-Value
Cardiovascular Events	9 (20)	5 (8)	0.063
Diagnostics	5 (11)	18 (30)	0.999
Infections	11 (24)	8 (13)	0.843
Flares	3 (7)	3 (5)	0.073
Neurological Event	6 (13)	2 (3)	0.806
Cancer	9 (20)	5 (8)	0.631
Kidney Transplant	2 (4)	1 (2)	0.999
C-section	0	1 (2)	0.999
Orthopaedic Surgery	3 (7)	8 (13)	0.367
Other Surgery	7 (15)	10 (16)	0.428

**Table 2:** Disease progression by accrual of new BILAG domains. Percentages of specific new BILAG manifestations are expressed as a fraction of the total number of patients with at least one new disease manifestation. BILAG–British Isles Lupus Assessment Group; CG–control group; PVG–post-vaccination group.

Patients with New Manifestations: *N* (%)	CG (*n* = 34/223)	PVG (*n* = 26/284)	*p*-Value
Cardiopulmonary domain	11 (32)	3 (12)	0.012
Haematological domain	12 (35)	0 (0)	<0.001
Musculoskeletal domain	3 (1)	17 (65)	0.010
Neuropsychiatric domain	1 (3)	0 (0)	0.440
Ophthalmic domain	1 (3)	0 (0)	0.440
Renal domain	4 (11)	3 (12)	0.705
Mucocutaneous domain	4 (11)	4 (15)	0.754

**Table 3:** Incidence Rates of chronic comorbidities of special interest per 100 person-month. CG–control group; PVG–post-vaccination group; ESRD–end stage renal disease.

Incidence Rates (Events/100 Person-Months)	CG (*n* = 223)	PVG (*n* = 284)	*p*-Value
Cancer	0.09	0.11	0.852
Cardiovascular disorders	0.35	0.07	<0.001
Cutaneous disorders	0.03	0.01	0.850
Fibromyalgia	0.05	0.00	0.082
Gonadal failure	0.01	0.00	0.900
Neurological disorders	0.09	0.00	0.007
Psychiatric disorders	0.08	0.00	0.016
Pulmonary disorders	0.03	0.05	0.961
Other autoimmune diseases	0.07	0.06	0.892
ESRD	0.03	0.06	0.633

**Table 4:** Pregnancies and pregnancy-related outcomes in CG and PVG (% over number of pregnancies). CG–control group; PVG–post-vaccination group.

Item	CG (*n* = 223)	PVG (*n* = 284)	*p*-Value
At least one pregnancy: *N* (%)	15 (7)	9 (3)	0.054
Number of pregnancies: *N*	16	11	-
Adverse foetal outcomes: *N* (%) *	3 (19)	2(18)	0.999
Adverse maternal events: *N* (%) *	6 (38)	3 (27)	0.897

* Percentage expressed as a fraction of the number of total pregnancies.

## Data Availability

The authors might share source data supporting this manuscript upon reasonable request to the corresponding author.

## Supplementary Materials

The following supporting information can be downloaded at https://www.mdpi.com/article/10.3390/vaccines13070735/s1, Table S1: disease characteristics according to BILAG domains among patients in the PVG at time of the first vaccine dose; Table S2: disease characteristics according to BILAG domains among patients in the CG at the start of follow-up; Table S3: short-term adverse events in the PVG.
