# Supplementary material for: Long Term Outcomes of Anti-COVID-19 Vaccines in Patients with Systemic Lupus Erythematosus: A Multicentre Study
Source: Vaccines (Basel). 2025 Jul 8;13(7):735. doi: 10.3390/vaccines13070735 (PMC12300575; doi:10.3390/vaccines13070735)
Supplement: Supplementary file 1 [file vaccines-13-00735-s001.zip › vaccines-3719927-supplementary.pdf]

Supplemental Material to

Long term outcomes of anti-COVID-19 vaccines in pa-tients with systemic lupus erythematosus: a multicentre study

Giovanni Benanti1,2^, Giuseppe Alvise Ramirez1,3^* Tommaso Schioppo4^, Lorenza Maria Argolini5^, Gabriella Moroni6, Grazia Bonelli7, Renato Alberto Sinico7, Federico Alberici8, Federica Mescia8, Luca Moroni1,3^, Gabriele Gallina1^, Biancamaria Venerandi1,3, Francesco Tamborini10, Chiara Bellocchi11,12^, Lorenzo Beretta11^, Roberto Caporali5,13^, Enrica Bozzolo 1,2^, Lorenzo Dagna1,3^, Maria Gerosa5,13^.

1 Unit of Immunology, Rheumatology, Allergy and Rare Diseases, IRCCS Ospedale San Raffaele, Milan Italy

2 Unit of General Medicine and Advanced Care, IRCCS Ospedale San Raffaele, Milan, Italy

3 Università Vita-Salute San Raffaele, Faculty of Medicine, Milan, Italy

4 Unit of Rheumatology, ASST Santi Paolo e Carlo, Milan, Italy

5 ASST Pini CTO, Lupus Clinic, Division of Clinical Rheumatology, 20122 Milan, Italy; lorenza.argolini@hotmail.it

6 Department of Biomedical Sciences, Humanitas University, IRCCS Humanitas Research Hospital, 20072 Milan, Italy; gabriella.moroni@hunimed.eu

7 Renal Unit, Department of Medicine and Surgery, Università degli Studi di Milano Bicocca and ASST-Monza, 20900 Monza, Italy; renato.sinico@unimib.it (R.A.S.); g.bonelli2@campus.unimib.it (G.B.)

8 Department of Medical and Surgical Specialties, Radiological Sciences and Public Health, University of Brescia, 25121 Brescia, Italy; federico.alberici@gmail.com (F.A.); federica.mescia@gmail.com (F.M.)

9 Department of Clinical Sciences and Community Health, Research Center for Adult and Pediatric Rheumatic Diseases, University of Milan, 20122 Milan, Italy; maria.gerosa@unimi.it (M.G.); roberto.caporali@unimi.it (R.C.)

10 Fondazione Ca' Granda IRCCS Ospedale Maggiore Policlinico Milano, Divisione di Nefrologia e Dialisi, 20122 Milan, Italy; francesco_tamborini@asst-pavia.it

11 Fondazione IRCCS Ca' Granda Ospedale Maggiore Policlinico di Milano, Referral Centre for Systemic Autoimmune Diseases, 20122 Milan, Italy; chiara.bellocchi@unimi.it (C.B.); lorberimm@hotmail.com (L.B.)

12 Department of Clinical Science of Community Health, Section of Internal Medicine, Università degli Studi di Milano, Milano, Italy

13 Department of Clinical Science of Community Health and Research Center for Adult and Pediatric Rheumatic Diseases, Università degli Studi di Milano, Milano, Lombardia, Italy

Supplemental Table S1: disease characteristics according to BILAG domains among patients in the PVG at time of the first vaccine dose

Items	Value
Number of subjects	284
Females: N (%)	252 (89)
Years of disease: median (IQR)	18 (11-27)
≤III Decade: N (%)	36 (13)
IV Decade: N (%)	56 (20)
V Decade: N (%)	58 (20)
VI Decade: N (%)	85 (30)
VII Decade: N (%)	36 (13)
>VII Decade: N (%)	13 (5)
Constitutional manifestations: N (%)	143 (50)
Mucocutaneous manifestations: N (%)	191 (67)
Neurological manifestations: N (%)	41 (14)
Musculoskeletal manifestations: N (%)	226 (80)
Cardiopulmonary manifestations: N (%)	68 (24)
Gastroenterological manifestations: N (%)	16 (6)
Ophtalmological manifestations: N (%)	11 (4)
Renal manifestations: N (%)	184 (65)
Haematological manifestations: N (%)	136 (48)

Abbreviations

BILAG: British Isles Lupus Assessment Group; IQR: interquartile range; PVG: post-vaccine group

Supplemental Table S2: disease characteristics according to BILAG domains among patients in the CG at the start of follow-up.

ITEMS	VALUE
Number of subjects	223
Females: N (%)	183 (88%)
Years of disease: median (IQR)	11 (6-21)
**≤III DECADE: N (%)**	39 (17%)
**IV DECADE: N (%)**	54 (24%)
**V DECADE: N (%)**	55 (25%)
**VI DECADE: N (%)**	48 (22%)
**VII DECADE: N (%)**	19 (9%)
**>VII DECADE: N (%)**	8 (4%)
**CONSTITUTIONAL MANIFESTATIONS: N (%)**	152 (68)
**MUCOCUTANEOUS MANIFESTATIONS: N (%)**	126 (57)
**NEUROLOGICAL MANIFESTATIONS: N (%)**	38 (17)
**MUSCULOSKELETAL MANIFESTATIONS: N (%)**	155 (70)
**CARDIOPULMONARY MANIFESTATIONS: N (%)**	64 (29)
**GASTROENTEROLOGICAL MANIFESTATIONS: N (%)**	8 (4)
**OPHTALMOLOGICAL MANIFESTATIONS: N (%)**	5 (2)
**RENAL MANIFESTATIONS: N (%)**	88 (39)
**HAEMATOLOGICAL MANIFESTATIONS: N (%)**	171 (77)

Abbreviations

BILAG: British Isles Lupus Assessment Group; CG: control group; IQR: interquartile range

Supplemental Table S3: short-term adverse events in the PVG

	First and second dose*	First dose	Second dose
Patients with at least one reaction: N	79	45	62
Symptoms and signs: N(%)			
Headache	10 (13)	6 (13)	5 (11)
Other neurological symptoms	3 (4)	3 (7)	2 (4)
Arthralgia	19 (24)	7 (16)	18 (40)
Myalgia	11 (14)	7 (16)	9 (20)
Fever	35 (44)	13 (29)	26 (58)
Local reaction	14 (18)	10 (22)	7 (16)
Gastrointestinal symptoms	11 (14)	4 (9)	8 (18)
Lymphadenopathy	4 (5)	2 (4)	3 (7)
Fatigue	16 (20)	11 (24)	9 (20)
Rash	8 (10)	5 (11)	3 (7)
Other	14 (18)	7 (16)	8 (18)

Abbreviations

PVG: post-vaccine group

* when applicable
